# Human milk oligosaccharide-sharing by a consortium of infant derived *Bifidobacterium* species

**DOI:** 10.1038/s41598-022-07904-y

**Published:** 2022-03-09

**Authors:** Clodagh Walsh, Jonathan A. Lane, Douwe van Sinderen, Rita M. Hickey

**Affiliations:** 1 Teagasc Food Research Centre, Moorepark, Fermoy, Co. Cork, P61C996 Ireland; 2 Health and Happiness Group, H&H Research, Cork, P61K202 Ireland; 3 APC Microbiome Ireland and School of Microbiology, University College Cork, Cork, Ireland

**Keywords:** Microbiome, Dietary carbohydrates, Applied microbiology, Symbiosis

## Abstract

Bifidobacteria are associated with a host of health benefits and are typically dominant in the gut microbiota of healthy, breast-fed infants. A key adaptation, facilitating the establishment of these species, is their ability to consume particular sugars, known as human milk oligosaccharides (HMO), which are abundantly found in breastmilk. In the current study, we aimed to characterise the co-operative metabolism of four commercial infant-derived bifidobacteria (*Bifidobacterium bifidum* R0071, *Bifidobacterium breve* M-16V, *Bifidobacterium infantis* R0033, and *Bifidobacterium infantis* M-63) when grown on HMO. Three different HMO substrates (2′-fucosyllactose alone and oligosaccharides isolated from human milk representing non-secretor and secretor status) were employed. The four-strain combination resulted in increased bifidobacterial numbers (> 21%) in comparison to single strain cultivation. The relative abundance of *B. breve* increased by > 30% during co-cultivation with the other strains despite demonstrating limited ability to assimilate HMO in mono-culture. HPLC analysis revealed strain-level variations in HMO consumption. Metabolomics confirmed the production of formate, acetate, 1,2-propanediol, and lactate with an overall increase in such metabolites during co-cultivation. These results support the concept of positive co-operation between multiple bifidobacterial strains during HMO utilisation which may result in higher cell numbers and a potentially healthier balance of metabolites.

## Introduction

Establishment of the infant gut microflora is a highly complex process, with feeding regime profoundly impacting on the development of neonate’s microbial gut ecology^1–3^. Human milk contains a high concentration of indigestible carbohydrates, known as human milk oligosaccharides (HMO), which can reach the large intestine in an intact form, where they can act as growth substrates for beneficial bacteria^4^. To date, it is estimated that over 200 different HMO structures exist, with overall levels in human milk varying from 5 to 23 g/L. All HMO consist of a lactose backbone which is decorated (see below) and/or elongated into a variety of oligomeric structures by the addition of β1-3- or β1-6-linked lacto-*N*-biose (Galβ1-3GlcNAc-, type 1 chain) or *N*-acetyllactosamine (Galβ1-4GlcNAc-, type 2 chain)^5,6^. HMO decoration is achieved by the addition of fucose residues (*e.g.*, 2′-fucosyllactose [2′-FL] and 3-fucosyllactose [3-FL]), or *N*-acetylneuraminic acid residues [NeuAc-also known as sialic acid] (*e.g.*, 3′-sialyllactose [3′-SL] and 6′-sialyllactose [6′-SL])^5,6^. Variability in the chemical and structural conformations of HMO is biologically relevant^7^.

These glycans serve as selective regulators of infant gut microbiota composition, and facilitate the development of a *Bifidobacterium*-dominant microbiota in breast-fed infants^8,9^. A high level of bifidobacteria in the gut microbiota of infants is believed to contribute to enhanced development of the immature gut immune system, and is associated with reduced levels of intestinal infections and diarrhea^10,11^. Due to their purported health-promoting effects, bifidobacteria have been incorporated into many functional foods and supplements as probiotic ingredients^12,13^. Many members of the genus *Bifidobacterium* are known to dedicate a relatively large proportion of their genome to carbohydrate utilization, reflecting their adaptation to metabolism of host-produced and/or host-ingested/dietary carbohydrates. Some of these genomes, particularly infant bifidobacterial species, display a clear adaptation to the nursing period when the diet of their host may be solely composed of (HMO-rich) breast milk^14–16^. One of the reasons as to why certain bifidobacterial species are such abundant and prevalent members of the (early) gut microbiota is due to their remarkable capacity to metabolize and consume (specific) HMO. In vitro growth experiments have revealed species-dependent utilization of individual HMO among *Bifidobacterium*^17,18^. These studies have enabled detailed profiling of (bifido)bacterial consumption of individual HMO and demonstrate specific and preferential oligosaccharide consumption by particular bifidobacterial strains^19–21^. However, in order to fully appreciate the role that HMO play in the creation of a *Bifidobacterium* dominant microbiota, consideration must be given to the cross-feeding relationships that exist between members of the infant microbiota^22^. A key determinant of microbial dynamics in the gut microbiota is represented by competition and sharing of nutrients. In the current study, we performed co-cultivation experiments involving four bifidobacterial strains when grown on HMO substrates to elucidate if, and to what extent, trophic interactions prevail among bifidobacteria when present in a competitive environment. Parameters intrinsic to efficient HMO metabolism (ability to support microbial growth, utility as substrates for fermentation, and production of organic acid metabolites) were measured to determine the impact of this syntrophic/competitive relationship. Unravelling HMO assimilation phenotypes and understanding the oligosaccharide structural complexity required to enrich specific beneficial bacterial communities allows for the development of targeted and personalised HMO-containing probiotic products with well-defined microbial, metabolic, and infant health outcomes.

## Results

### *Bifidobacterium* strains display divergent and strain-specific growth phenotypes, with enhanced growth performance during co-cultivation

**Figure 1:**
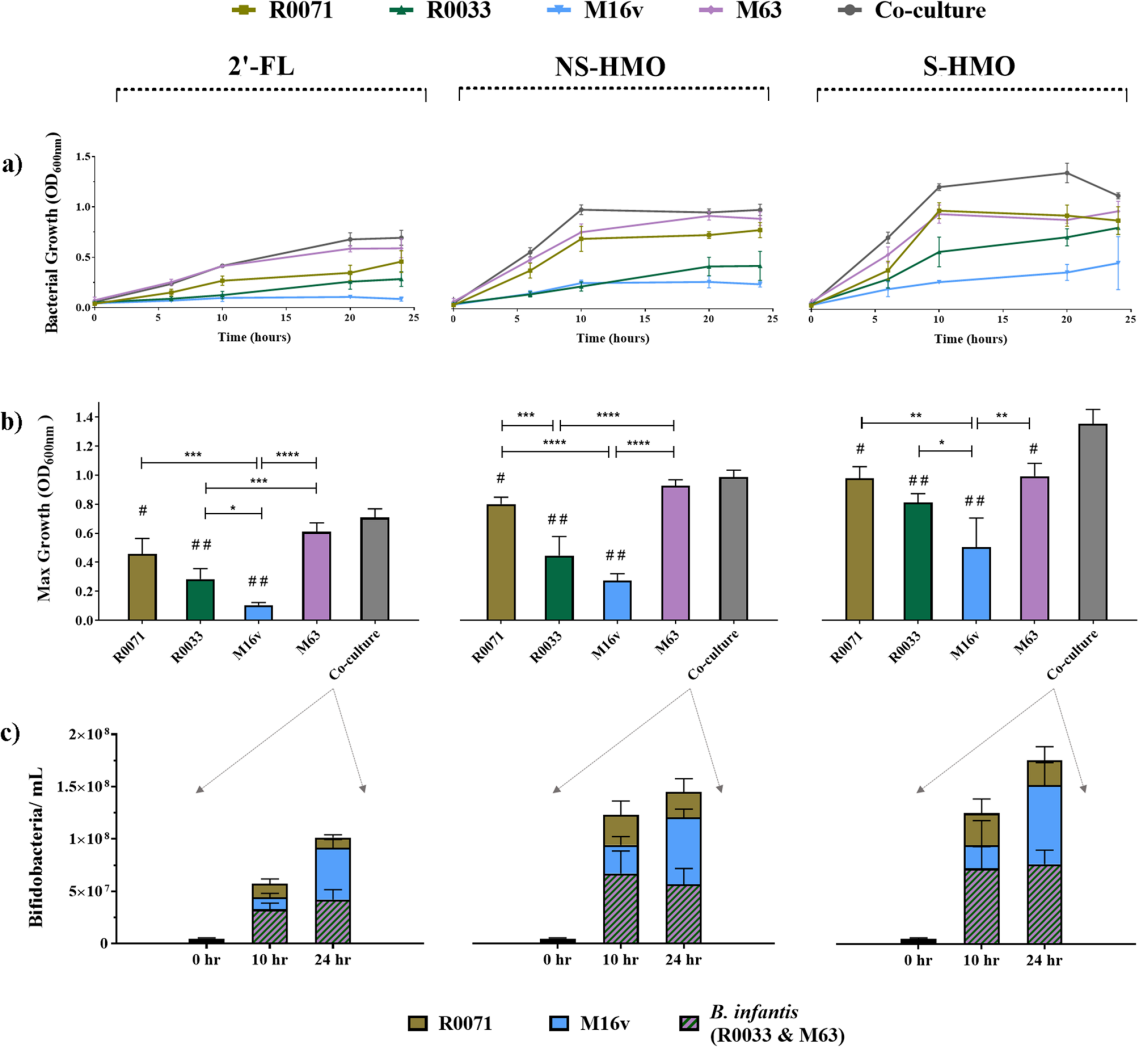
Growth profiles of bifidobacterial strains as mono-cultures and as a four strain co-culture with the following HMO as the sole carbon source: 2’-FL [1.2 g/L], model of NS-HMO [3.8 g/L] and model of S-HMO [5 g/L]. (**a**) Growth of strains in mono- and co-culture at 0 h, 6 h, 10 h, 20 h and 24 h. (**b**) Maximum levels of growth reached by the strains in mono- and co-culture (**c**) Growth (bifidobacteria/mL) of subspecies within the four strain co-culture at 0 h, 10 h and 24 h. Strains within co-culture were quantified using qPCR with primers targeting the 16 s gene region of each subspecies. OD600nm = Optical density at 600 nm. R0071 = *Bifidobacterium bifidum* R0071, R0033 = *Bifidobacterium infantis* R0033, M-16V* = Bifidobacterium breve* M-16V, M-63 = *Bifidobacterium infantis* M-63. Analysis was calculated using technical triplicate data from biological triplicate experiments and data are means  + /−SD. Univariate analysis of variance (ANOVA) and post-hoc Tukey tests were performed to determine the significant differences between the groups (#  =  *p* < 0.05, ##  = * p* < 0.001 vs. co-culture) (* = *p* < 0.05, ** =  *p* < 0.01, *** =  *p* < 0.001, **** =  *p* < 0.0001 between other strain combinations).

Growth profiles of *Bifidobacterium* *bifidum* Rosell®-71 (R0071), *Bifidobacterium* *infantis* Rosell®-33 (R0033), *Bifidobacterium* *breve* M-16V, and *Bifidobacterium* *infantis* M-63 cultivated in mono-culture on HMO-supplemented media were evaluated and compared to the growth profiles of the strains when grown in a four strain co-culture on the same carbohydrate sources (Fig. 1a,b,c). The same experiments were performed on MRS without any carbohydrate source, revealing, as expected, an absence of appreciable growth (showing that carbohydrate free MRS [cfMRS] is unable to support bacterial growth above an OD_600nm_ of 0.1). HMO was successfully separated and purified from pooled breast milk by gel-filtration chromatography. Preliminary screenings indicated that, during size exclusion on Bio-Gel polyacrylamide beads, lactose and 2′-FL tended to fractionate together in the same eluates. Here, this apparent drawback was exploited in order to generate multiple HMO powders which mimicked physiologically relevant oligosaccharide blends. All fractions with high levels of lactose and 2′-FL were eliminated from the final HMO-enriched blend to give a powder more representative of non-secretor HMO (designated NS-HMO). Although the most abundant α1,2-fucosylated HMO was eliminated from the powder, the preparation contained other H-antigen containing HMOs such as difucosyllactose (DFL), lacto-*N*-fucopentaose I (LNFP I), and lacto-*N*-difucohexaose I (LNDFH I) (Supplementary Fig. S1). The 2′-FL which had been eliminated during the size exclusion process was then resubstituted to give a powder representative of secretor HMO (designated S-HMO) (Supplementary Fig. 1). In total, therefore, three HMO substrates were assessed in this study: 2′-FL (at 1.2 g/L), NS-HMO (at 3.8 g/L), and S-HMO (at 5 g/L) (compositions of the HMO powders are outlined in Supplementary Fig. S1).

Most notably, significant growth differences were observed between the two *B. infantis* strains (Fig. 1a,b,c) despite these strains showing similar growth in MRS media with glucose and lactose as sole carbohydrate sources (data not shown). When grown on 2′-FL, strain M-63 demonstrated a significantly higher maximum OD_600_ (*p* = 0.0004) and higher growth rate during the exponential phase (*p* =  < 0.0001) of growth, when compared to strain R0033 (Fig. 1). Similar findings were noted during growth on NS-HMO (Fig. 1). Although R0033 and M-63 both grew vigorously on S-HMO (reaching final OD_600_ values of 0.81 and 0.99, respectively), the observed growth rate of M-63 during the exponential phase was significantly higher than that of R0033 (*p* = 0.0023) (Fig. 1a). M-63, however, did display comparable growth profiles to R0071. *Bifidobacterium* *bifidum* R0071 reached a maximum OD_600_ of 0.81 and 0.98 on NS-HMO and S-HMO, respectively (Fig. 1).

*Bifidobacterium* *breve* M-16V did not exhibit any significant growth on 2′-FL (Fig. 1) yet reached an OD_600_ of 0.27 and 0.51 when grown on NS-HMO and S-HMO, respectively (Fig. 1b). Co-cultivation of this strain with R0071, R0033, and M-63 on 2′-FL (*p* < 0.0001), NS-HMO (*p* = 0.0033) or S-HMO (*p* = 0.0002) resulted in significant increases of M-16V cell numbers when compared to its growth in mono-culture (Fig. 1c). Overall, co-cultivation of all four *Bifidobacterium* strains together on S-HMO resulted in significantly higher cell numbers when compared to single strain cultivations (Fig. 1b). During growth on S-HMO co-cultivation resulted in a 27.7% increase in cell numbers when compared to the highest mono-culture fermentation of R0071 (*p* = 0.0096) (Fig. 1b). Quantitative PCR analysis targeting the 16S rRNA region allowed for quantification of cell numbers of each species within the co-culture, but did not distinguish between the two *B. infantis* strains. Nonetheless, qPCR analysis provided insights into the microbial dynamics in the co-culture system and revealed significant shifts in microbial distribution across the fermentation period. Despite significant increases in bacterial cell numbers, no change in the relative abundance of species within the co-culture occurred from 0 to 10 h. At the initiation of fermentation on S-HMO the co-culture contained 1.23 (± 0.41) × 10^6^ bifidobacteria/mL *B. bifidum*, 1.38 (± 0.39) × 10^6^ bifidobacteria/mL *B. breve* and 2.61 (± 0.46) × 10^6^ bifidobacteria/mL *B. infantis*. No significant change had occurred in this population distribution by late exponential phase (10 h) with each species within the co-culture displaying similar growth rates (µ) and doubling times (T_d_). During this period (0–10 h) *B. bifidum, B. breve,* and *B. infantis* grew at a rate of 0.32 ± 0.01, 0.3 ± 0.02, and 0.33 ± 0.04 h^−1^ respectively. However, between exponential (10 h) and stationary phase (24 h), significant shifts in microbial distribution were observed. While bacterial cell numbers of *B. bifidum* (*p* = 0.8476) and *B. infantis* (*p* = 0.9419) remained constant between 10 and 24 h fermentation, growth of *B. breve* (*p* = 0.0010) persisted. Therefore, after 24 h growth on S-HMO, the co-culture contained 2.63 (± 0.95) × 10^7^ bifidobacteria/mL *B. bifidum*, 7.57 (± 2.14) × 10^7^ bifidobacteria/mL *B. breve* and 7.63 (± 1.36) × 10^7^ bifidobacteria/ mL *B. infantis*. qPCR analysis showed an overrepresentation of *B. breve* at 24 h when compared to bifidobacterial numbers of *B. bifidum* and *B. infantis.*

Further insights into growth parameters of strains during co-cultivation were provided by performing co-culture growth of *B. bifidum* R0071 with (a) *B. infantis* R0033 on DFL (Supplementary Fig. S2) and (b) *B. breve* M-16V on 3’-SL (Supplementary Fig. S3). In both instances, co-cultivation resulted in significantly higher cell numbers in comparison to mono-culture growth of strains on the same carbohydrates. During growth on DFL, single-strain cultures of R0071 and R0033 achieved maximum growth of 2.02 (± 0.36) × 10^8^ CFU/mL and 1.98 (± 0.57) × 10^8^ CFU/mL respectively (Supplementary Fig. S2). When these strains were grown in co-culture on the same carbohydrate, the culture contained 3.47 (± 0.35) × 10^8^ CFU/mL (*p* < 0.0001 vs. both mono-culture situations) with *B. bifidum* R0071 occupying 22.08 ± 4.16% of total cell numbers (Supplementary Fig. S2).

*Bifidobacterium* *bifidum* R0071 was shown to grow well on 3′-SL in mono-culture reaching a viable count of 1.89 (± 0.51) × 10^8^ CFU/mL after 24 h incubation. As per its growth on more complex HMO blends, *B. breve* M-16V demonstrated limited ability to grow on 3′-SL in mono-culture (Supplementary Fig. S3). However, significant growth of this strain in 3′-SL was observed when cultured in combination with *B. bifidum* R0071 (Supplementary Fig. S3). The co-culture of R0071 and M-16V contained 3.11 (± 0.86) × 10^8^ CFU/ mL after 24 h growth on 3′-SL (*p* < 0.0001 vs. either of the mono-culture situations) with *B. breve* M-16V accounting for 86.89 ± 4.84% of the total cell number proportion (Supplementary Fig. S3).

### Detailed analysis of bifidobacterial degradation of oligosaccharides confirms strain-specific and structurally selective consumption of HMO

**Figure 2:**
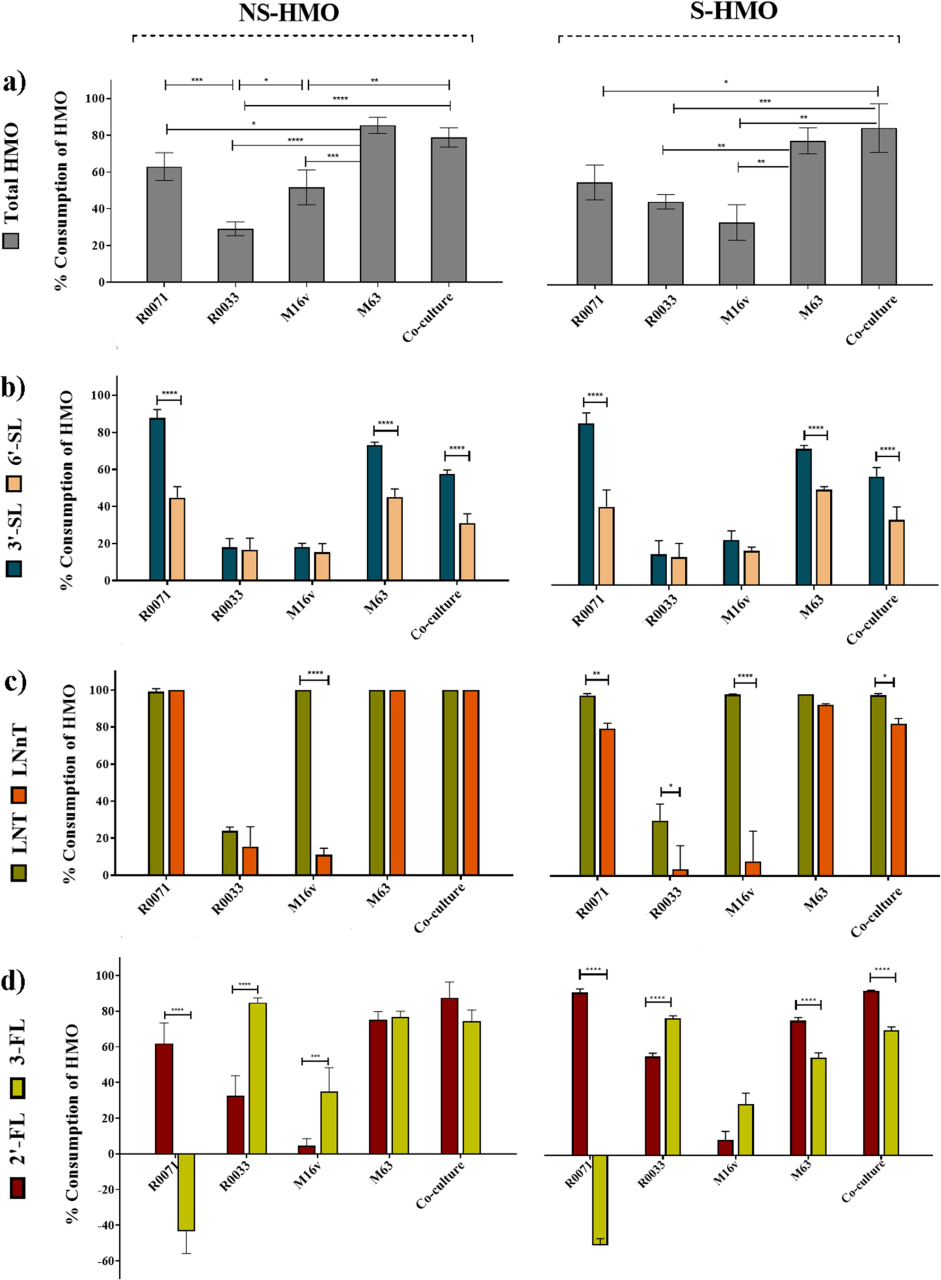
Percentage consumption of oligosaccharides after 10 h of bifidobacterial fermentation on a model of NS-HMO and S-HMO versus the uninoculated control. The concentrations of oligosaccharide in culture were determined by HPAEC-PAD. The eluted sugars were identified from their elution times relative to those of external standards (lactose, 3-fucosyllactose [3-FL], 2′-fucosyllactose [2′-FL], lacto-*N*-neotetraose [LNnT], lacto-*N*-neohexaose [LNnH], lacto-*N*-tetraose [LNT], lacto-*N*-hexaose [LNH], sialic acid, sialyllacto-*N*-tetraose a [LSTa], sialyllacto-*N*-tetraose c [LSTc], 6′-siallylactose [6′-SL], 3′-siallylactose [3′-SL], disialyllacto-*N*-tetraose [DSLNT]) (**a**) Consumption of total HMO, (**b**) Consumption of the siallylactoses, 3′-SL and 6’-SL, (**c**) Consumption of the tetraoses, LNT and LNnT and (**d**) Consumption of the fucosyllactoses, 2′-FL and 3-FL. R0071 = *Bifidobacterium bifidum* R0071, R0033 = *Bifidobacterium infantis* R0033, M-16V = *Bifidobacterium breve* M-16V, M-63 = *Bifidobacterium infantis* M-63. Analysis was calculated using technical duplicate data from biological triplicate experiments and data are means + /− SD. Univariate analysis of variance (ANOVA) and post-hoc Tukey tests were performed to determine the significant differences between the groups (* = *p* < 0.05, ** = *p* < 0.01, *** = *p* < 0.001, **** = *p* < 0.0001).

Glycoprofiling of bifidobacterial strains using HPLC methods revealed significant variations in oligosaccharide utilization (Supplementary Fig. S4). Typically, higher levels of HMO consumption were observed in cultures with greater cell numbers. For instance, the numerical dominance of *B. infantis* M-63 over its subspecies counterpart *B. infantis* R0033 was similarly reflected in these strains’ glycoprofile patterns. In comparison to M-63, significantly higher oligosaccharide amounts remained in the culture after 10h growth of R0033 in 2′-FL (*p* = 0.0093), NS-HMO (*p* < 0.0001), or S-HMO (*p* = 0.0091) (Supplementary Fig. S4). During growth on S-HMO, M-63 consumed 78.8% of the total HMO, while R0033 only assimilated 45.5% of available HMO (Fig. 2a). HPLC analysis of the culture supernatant showed that the majority of 2′-FL (79.2%), 3-FL (57.4%), 3′-SL (74.1%), 6′-SL (51.9%), LNT (100%) and LNnT (94.2%) was degraded after 10 h fermentation with M-63 (Fig. 2). Overall, glycoprofiling of M-63 culture supernatants indicated that LNT and LNnT are preferred substrates for this *B. infantis* strain (Fig. 2c). On the other hand, R0033 exhibited limited consumption of LNT (30.4%) and LNnT (3.7%), as well as 3′-SL (16.7%) and 6′-SL (15.1%). Instead, the fucosyllactoses served as preferred nutrients for R0033, with 58.1% of 2′-FL and 80.6% of 3-FL hydrolysed by late exponential phase (Fig. 2d). Notably, during fermentation on S-HMO, R0033 preferentially consumed 3-FL rather than its predominant structural isomer 2′-FL (*p* < 0.0001) (Fig. 2d). Again, this diverges from the saccharolytic activity observed in *B. infantis* M-63, with this strain preferentially consuming 2′-FL over 3-FL (*p* = 0.0058) (Fig. 2d). Further structure-specific consumption of HMO isomers was observed for M-63, with significantly higher preference for 3′-SL over 6′-SL (*p* < 0.0001) (Fig. 2b).

In comparison to M-63, *B. breve* M-16V demonstrated limited utilization of most HMO structures. During growth on S-HMO, M-16V consumed 24.4% of 3′-SL, 18.6% of 6′-SL, 8.9% of 2′-FL, 30.1% of 3-FL, and 8.1% of LNnT (Fig. 2). LNnT represents the core structure of type 2 HMO, whereas LNT represents the core structure of type 1 HMO. M-16V demonstrated significant consumption of the type 1 HMO LNT (Fig. 2c). One hundred percent of available LNT was metabolised by M-16V after 10 h fermentation (Fig. 2c).

*Bifidobacterium* *bifidum* R0071 readily assimilated most HMO structures (Fig. 2 and Supplementary Fig. S4). As per M-16V consumption of the tetrasaccharides, R0071 preferentially consumed type 1 LNT rather than type 2 LNnT (99.3% consumption vs. 80.9%, *p* = 0.0023) (Fig. 2c). Similarly, R0071 favoured consumption of lacto-*N*-hexaose (LNH) (which harbours the type 1 moiety LNB) over lacto-*N*-neohexaose (LNnH) (which harbours the type 2 moiety *N*-acetyllactosamine) (*p* < 0.0001). Moreover, R0071 demonstrated preferential consumption of 3′-SL over 6′-SL (88.1% consumption vs. 42.6% consumption, *p* < 0.0001) (Fig. 2b). One of the most notable observations made during oligosaccharide glycoprofiling of *B. bifidum* R0071 was the significant increase in the levels of 3-FL following fermentation on NS-HMO (increase of 36.76% of 3-FL) and S-HMO (increase of 46.09% of 3-FL) (Fig. 2d). No other strain combination resulted in increased levels of 3-FL. Increased levels of 3-FL were also observed during growth of *B. bifidum* R0071 with DFL as sole carbohydrate source (Supplementary Fig. S2). After 10 h growth of *B. bifidum* R0071, 68.03% DFL had been consumed which corresponded to the release of 232.1 ± 26.1 mg/L of 3-FL. HPAEC-PAD analysis indicated that this increased level of 3-FL did not persist throughout fermentation, with only 17.3 mg/mL 3-FL remaining after 24 h growth of *B. bifidum* R0071 (decrease of 92.47% vs. 10 h) (Supplementary Fig. 2). During co-cultivation of R0071 with *B. infantis* R0033 on the same DFL-containing media, detectable levels of DFL and 3-FL were found in the culture after 10 h, but significantly less when compared to mono-culture growth of R0071 (Supplementary Fig. S2). After 10 h co-culture of R0071 and R0033, the concentration of DFL had decreased by 92.01% (*p* < 0.0001 vs. R0071 mono-culture) with 21.9 mg/mL of 3-FL detected in the culture (*p* < 0.0001 vs. R0071 mono-culture). After 24 h co-cultivation of R0071 and R0033, neither DFL nor 3-FL were detected in the culture supernatant (Supplementary Fig. S2).

Monosaccharide analysis using HPAEC-PAD revealed that large quantities of sialic acid are released during growth of *B. bifidum* strain R0071 on NS-HMO (118.8 mg/L) and S-HMO (134.6 mg/L) (Supplementary Fig. S5). Significant quantities of sialic acid (24 mg/L in NS-HMO and 30 mg/L in S-HMO) were also detected in the M-63 cell-free supernatant after 10 h growth (Supplementary Fig. S5). The levels of sialic acid produced following fermentation with *B. bifidum* R0071 were in stoichiometric accordance with levels of depleted sialylated HMO. In S-HMO, R0071 assimilated 0.46 ± 0.04 mM sialylated HMO, and released 0.44 ± 0.03 mM sialic acid (molar ratio of depleted sialylated HMO to sialic acid produced = 1.07 ± 0.13). This molar balance of depleted sialylated HMO and production of sialic acid was not observed during M-63 fermentation. In S-HMO, *B. infantis* M63 consumed 0.46 ± 0.04 mM sialylated HMO but produced only 0.1 ± 0.02 mM sialic acid (molar ratio of depleted sialylated HMO to sialic acid produced = 4.82 ± 1.03).

Glycoprofiling of *B. bifidum* R0071 and *B. breve* M-16V cultures with 3′-SL as sole carbohydrate source was performed to assess whether the sialic acid released by *B. bifidum* R0071 is utilised by other strains in a co-culture system. In accordance with its growth on more complex HMO blends, M-16V was incapable of utilising 3′-SL (Supplementary Fig. S3). Mono-culture growth of R0071 resulted in the consumption of 60.51% 3′-SL at 10 h and 100% 3′-SL at 24 h which corresponded to a release of 159.3 mg/mL (10 h) and 234.6 mg/mL (24 h) of sialic acid (Supplementary Fig. S3). Co-cultivation of the two strains resulted in similar depletions of 3′-SL (69.91% at 10 h, 100% at 24 h) but significantly lower amounts of sialic acid (41.7 mg/mL at 10 h, 0 mg/mL at 24 h) (Supplementary Fig. S3).

Across all strains and carbohydrate combinations assessed in this study, levels of lactose after 10 h growth were below the limit of detection. All residual lactose found in the HMO powders at initiation of fermentation (approximately 130 mg/L) and any lactose released from the breakdown of HMO into its constituent building blocks were consumed by the strain(s) by 10 h.

### Production of metabolic end-products is dependent on strain combination and HMO substrate

**Figure 3:**
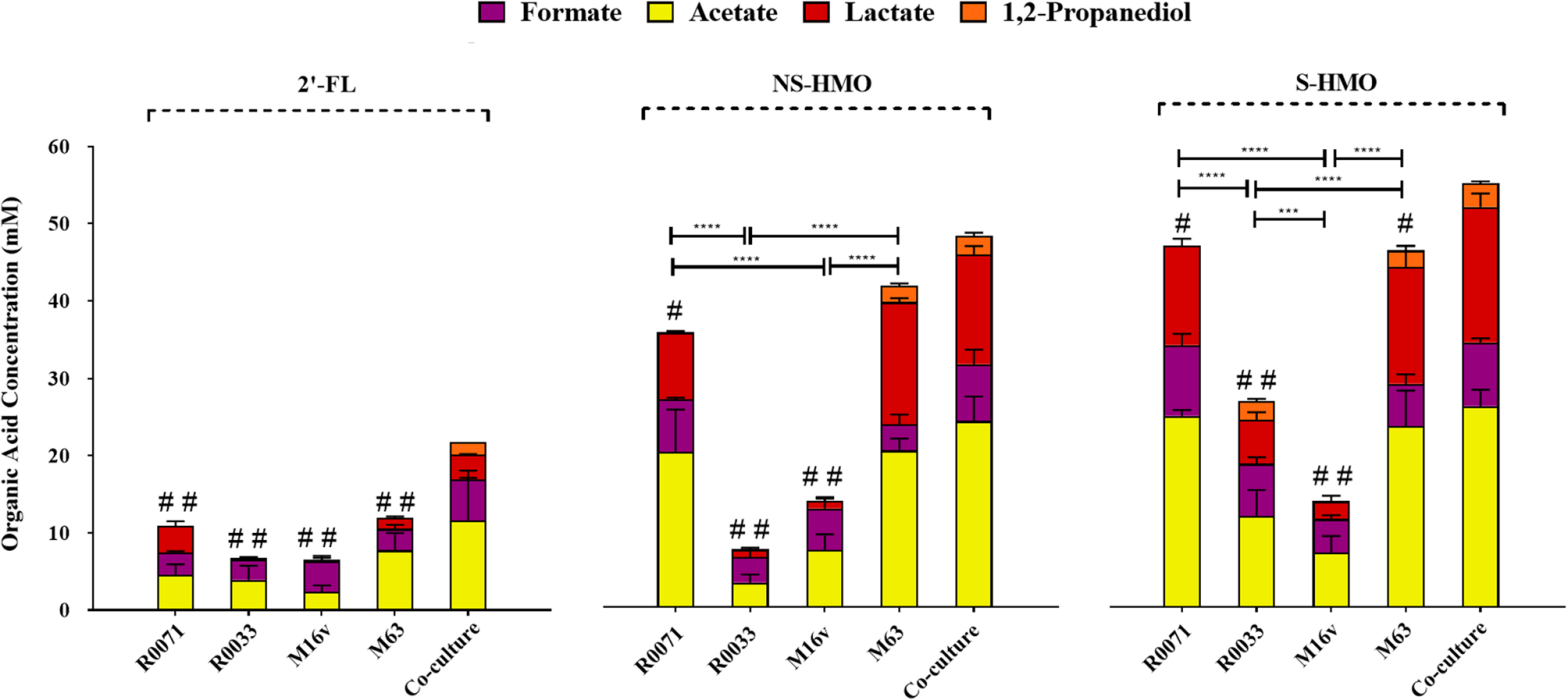
Levels of organic acids and 1,2-propanediol (1,2-PD) produced after 10 h of growth of the bifidobacterial strains as mono-cultures and as a four strain co-culture. The following HMO were used as the sole carbon source: 2′-FL [1.2 g/L], model of NS-HMO [3.8 g/L] and a model of S-HMO [5 g/L]. Levels of acetic acid, formic acid, and lactic acid were quantified using HPLC coupled with an RI detector. Levels of 1,2-PD were quantified using GC–MS. R0071 = *Bifidobacterium* *bifidum* R0071, R0033 = *Bifidobacterium* *infantis* R0033, M-16V = *Bifidobacterium* *breve* M-16V, M-63 = *Bifidobacterium* *infantis* M-63. Analysis was calculated using technical duplicate data from biological triplicate experiments and data are means  + /− SD. Univariate analysis of variance (ANOVA) and post-hoc Tukey tests were performed to determine the significant differences between the groups (# = *p* < 0.05, ## = *p* < 0.001 vs. co-culture) (* = *p* < 0.05, ** = *p* < 0.01, *** = *p* < 0.001, **** = *p* < 0.0001 between other strain combinations).

To assess the metabolic consequences of bifidobacterial growth on HMO, levels of the possible fermentative end-products acetate, lactate, formate, and 1,2-propanediol (1,2-PD) were evaluated. The absolute concentrations of these metabolites following 10 h fermentation are presented in Fig. 3 and Supplementary Table S1. The central metabolic pathways employed in *Bifidobacterium* for the production of these organic acids is shown in Supplementary Fig. S6. Measurable levels of acetate, lactate, and formate were detected across all strain/HMO combinations, albeit with significant variations (Fig. 3). Consistent with increased bifidobacterial numbers detected during growth analysis, co-culture fermentation of the four bifidobacterial strains resulted in significantly higher total metabolites compared to mono-culture fermentations (Fig. 3 and Supplementary Table S1). In comparison to single-strain cultivation with M63, metabolite production increased by 45.4%, 13.6%, and 15.9% during co-cultivation on 2′-FL, NS-HMO, and S-HMO, respectively. On the whole, acetate, and lactate levels were lowest during metabolism of 2′-FL and highest in cultures grown in S-HMO. Production of acetate ranged from 2.36 mM during M-16V growth on 2′-FL to 25.6 mM during co-culture metabolism of S-HMO. Likewise, lowest concentrations of lactate were detected following M-16V growth on 2′-FL (0.21 mM) and highest during co-culture growth on S-HMO (17.29 mM). Notable variances were observed between metabolic profiles of the two *B. infantis* strains. *B. infantis* M-63 produced significantly higher amounts of acetate when compared with R0033 during metabolism of 2′-FL (7.76 mM vs. 3.94 mM, *p* = 0.0233), NS-HMO (19.87 mM vs. 3.08 mM, *p* < 0.0001), and S-HMO (23.11 mM vs. 11.55 mM, *p* < 0.000).

Conversely, variability in formate production between strains was less pronounced, ranging from 2.68 to 9.1 mM (Fig. 3 and Supplementary Table S1). Overall, it is presumed that the concentrations of organic acids produced during bifidobacterial fermentation of carbohydrates are positively correlated with bacterial cell numbers. Pearson correlation and linear regression analysis was performed to assess the relationship between bacterial biomass and the subsequent yields of acetate, lactate, and formate (data not shown). Findings indicate that, across all three HMO substrates, acetate and lactate concentrations are positively correlated with bacterial cell numbers. However, no correlation between bacterial cell numbers and levels of organic acids were noted in the case of formic acid production.

Quantitative analysis of metabolite 1,2-PD using GC–MS revealed that production of 1,2-PD only occurred in the presence of the *B. infantis* strains and not during either *B. bifidum* or *B. breve* mono-culture fermentations, irrespective of the carbohydrate source (Fig. 3 and Supplementary Table 1). During growth on 2’-FL as sole carbon source, 1,2-PD production was only observed after fermentation with the four strain *Bifidobacterium* co-culture, with 1.7 mM detected after 10 h growth (Fig. 3). Co-culture fermentation of NS-HMO and S-HMO also resulted in significant quantities of 1,2-PD being produced (2.4 mM and 3 mM, respectively) (Fig. 3). 1,2-PD was also detected in *B. infantis* R0033 culture supernatants on S-HMO (2.4 mM), and in M-63 culture supernatants when grown on NS-HMO (2.1 mM) and S-HMO (2.1 mM) (Fig. 3).

Molar ratios of organic acids were determined to provide further insights into the metabolic trajectories during bifidobacterial HMO fermentation. These ratios are given in Table 1. For every 2 mol of hexose that enter into the *Bifidobacteriaceae*-specific bifid shunt metabolic pathway, 3 mol of acetic acid and 2 mol of lactic acid are produced (Supplementary Fig. S6). The theoretical ratio of 1.5 acetate: lactate was observed during *B. bifidum* R0071, *B. infantis* M-63, and co-culture fermentation across all HMO substrates (Table 1). In contrast, growth of *B. infantis* R0033 and *B. breve* M-16V on 2′-FL as sole carbon source significantly increased the acetate to lactate ratio to 7.4 ± 1.8 and 9.9 ± 3.4 (Table 1). Findings show that *B. infantis* R0033 and *B. breve* M-16V metabolism was characterized by an increase in formic acid production (relative to acetic and lactic acid production) (Table 1). A significant increase in the ratios of formate: acetate and formate: lactate was observed for R0033 and M-16V when compared to all other strain combinations (Table 1).

**Table 1:** Ratios of organic acids after 10 h fermentation.

	Acetate: lactate			Formate: acetate			Formate: lactate		
	2'-FL	NS-HMO	S-HMO	2'-FL	NS-HMO	S-HMO	2'-FL	NS-HMO	S-HMO
*Bbif* R0071	1.1 ± 0.2	2.3 ± 0.6	1.9 ± 0.2	0.6 ± 0.1	0.4 ± 0.1	0.4 ± 0.1	0.9 ± 0.2	0.8 ± 0.3	0.7 ± 0.1
*BInf* R0033	7.4 ± 1.8*	3.4 ± 1.7	2.1 ± 0.4	0.9 ± 0.2*	1.1 ± 0.2*	0.7 ± 0.1*	17.9 ± 6*	3.6 ± 1.9*	1.2 ± 0.2*
*Bbrev* M-16V	9.9 ± 3.4*	8 ± 3.2*	3.2 ± 0.8*	1.9 ± 0.4*	0.8 ± 0.2	0.7 ± 0.1*	26.1 ± 9.4*	6.2 ± 2.1*	2 ± 0.6*
*Binf* M-63	4.9 ± 2	1.3 ± 0.1	1.6 ± 0.6	0.4 ± 0.1	0.2 ± 0.1	0.2 ± 0.2	2.1 ± 0.7	0.2 ± 0.1	0.4 ± 0.2
4Bif co-culture	3.6 ± 1.7	1.7 ± 0.1	1.5 ± 0.1	0.5 ± 0.3	0.3 ± 0.1	0.3 ± 0.1	1.6 ± 0.3*	0.5 ± 0.1	0.5 ± 0.1

*p value < 0.05 versus theoretical ratio (acetate: lactate = 1.5, formate: acetate = 0.4, formate: lactate = 0.6).

## Discussion

This study aimed to characterise the metabolic capability and co-operative metabolism of a community of four infant *Bifidobacterium* strains when grown on HMO. Since formula-fed infants lack exposure to these potentially health-promoting oligosaccharides and microbes, delivery of HMO and probiotic bifidobacteria in infant formula or supplements remains a priority^23^. The four strains included in this study were selected based on their proven efficacy in clinical studies^24–26^. For instance, formula-fed infants receiving a probiotic supplement containing *B. bifidum* R0071 and *B. infantis* R0033 in addition to a *Lactobacillus* strain, maintained higher faecal SIgA levels at the end of the four-week treatment period^24^. *Bifidobacterium* *breve* M-16V is also a commonly used probiotic strain in infants and has been shown to offer potential in protecting infants from developing the necrotising enterocolitis (NEC) and allergic diseases (reviewed by Wong et al. 2019^25^). A mixture containing *B. infantis* M63 and two other probiotic strains (one being M-16V) was associated with better control of functional abdominal pain and improved quality of life when compared to the placebo in children with IBS^26^.

A key factor in determining microbial dynamics in the microbiota is the sharing and/or competition for nutrients^27^. In this context, microbe–microbe interactions can either positively or negatively influence the fitness of organisms present. The current study demonstrates that, during growth on HMO, cross-feeding interactions between multiple bifidobacterial strains increases overall cell numbers. This increase was hallmarked by notable shifts in microbial distribution from exponential to stationary phase. We found that under certain conditions (i.e., when fermentable carbohydrates were readily available) these *Bifidobacterium* strains stably coexist and no (sub)species appeared to have a considerable competitive advantage. However, over time, consumption of limiting nutrients and release of acidic metabolites appeared to shape the course of competition, thereby altering the relative abundance of species in the culture. No increase in bacterial cell numbers was observed for *B. bifidum* or *B. infantis* from exponential to stationary phases of growth during co-cultivation. Any increase in growth was due to continued proliferation of *B. breve*. This indicates that, despite demonstrating limited capacity to metabolize HMO in pure culture, the ability of M-16V to act as a ‘scavenger species’ and potential higher resistance to acidic conditions allows the strain to persist at the lowest resource level and become the competitive dominant in a shared environment. These findings appear to reflect the distribution of bifidobacterial species in the gut of breast-fed infants. Prototypical HMO-consumers *B. bifidum* and *B. infantis*, are often detected in low numbers in the feces of breast-fed infants, while *B. longum* and *B. breve* are regularly found as the dominant species in infant stools, even though they demonstrate minimal growth on HMO in vitro^28^.

Generally, it is assumed that *B. breve* has limited ability to metabolise complex HMO structures, but can grow on mono- and di-saccharide HMO degradants^29^. More recent studies have identified *B. breve* strains capable of metabolising a greater repertoire of HMO structures, including LNT and LNnT^30^, and those decorated with fucose and sialic residues^21^. Thus, the ability of *B. breve* to assimilate particular HMO is strain-dependent. In mono-culture *B. breve* M-16V was shown to exhibit rather poor growth on all assessed HMO substrates. Glycoprofiling revealed that M-16V consumed minimal amounts of 3-FL, 2′-FL, 3′-SL, 6′-SL, and LNnT, corroborating findings from previous in vitro growth studies^31^. We demonstrate here that M-16V is an efficient consumer of lacto-*N-*tetraose (LNT), the central moiety of Type I HMO. Similar selective consumption patterns have also been observed in vivo during probiotic administration of *B. breve* M-16V to premature breast-fed infants^19,32^.

Growth and sugar analysis presented here revealed notable strain-level differences in the ability of the two *B. infantis* probiotic strains to utilize HMO. *Bifidobacterium* *infantis* R0033 exhibited minimal growth on HMO and only demonstrated significant consumption of the fucosyllactoses after 10h of growth. In contrast, the probiotic strain *B. infantis* M-63 showed high levels of growth on 2′-FL, NS-HMO, and S-HMO. HPLC analysis of the resultant supernatant showed that the majority of HMO structures are degraded after 10 h fermentation with M-63, with preferential consumption of LNT and LNnT. Sakanaka et al. (2020), via genome data mining, found conservation of HMO-related genes (including GHs) across *B. infantis* to be very high^33^. It is assumed, therefore, that all *B. infantis* strains behave in the same manner when utilizing HMO. However, a recent study has reported genetic differences in HMO utilization clusters among *B. infantis* strains which has significant implications on fitness and performance of *B. infantis* strains in culture and in the infant gut^34^. Comparative genome analysis of commercial *B. infantis* probiotics revealed two main variants; one which harboured the full repertoire of HMO utilization genes and another variant which lacks an ABC-transporter needed for binding of core HMO structures (such as LNT and LNnT)^34^. We hypothesise that similar genotypic variations exist between *B. infantis* strains M-63 and R0033 which may have implications for their fitness and survival in the infant gut.

In contrast to the intracellular consumption strategies used by *B. infantis*, *B. bifidum* employs cell wall-anchored secretory glycosidases which hydrolyse the HMO extra-cellularly releasing mono- and di-saccharides, which are then internalized for metabolism and growth^19,33^. Here, we report that *B. bifidum* R0071 grows well on HMO and readily assimilates most HMO structures including type I structures, type II structures and sialylated structures. R0071 demonstrated preference for consumption of 3′-SL over 6′-SL. Both these structures are trisaccharide HMO that have the same molecular composition and only differ in the attachment of sialic acid residues around the lactose core. These findings highlight that the specific structure of the HMO isomers is biologically relevant. *Bifidobacterium* *bifidum* R0071 also demonstrated substantial consumption of 2′-FL, with the majority of the substrate degraded by late exponential phase. Remarkably, dramatic increases in the levels of 3-FL were observed during R0071 fermentation of HMO. To the best of our knowledge, this is the first time that an increase in the level of 3-FL during bifidobacterial growth on HMO has been reported. We speculate that this increase in 3-FL can be accounted for by differences in the catalytic activity of 1,2-α-L-fucosidase (AfcA) and 1,3/4-α-L-fucosidase (AfcB) as previously described^35^. It is plausible to hypothesise that the rapid action of AfcA cleaves the α-1,2 linked fucose from DFL to release 3-FL into the supernatant, which subsequently remains in the culture due to the low catalytic activity of AfcB. To test this hypothesis, growth experiments on DFL alone were performed. Glycoprofiling of culture supernatants confirmed that 3-FL was released during growth of R0071. However, this increase in 3-FL levels was found to be transient, suggesting that the AfcB harboured by R0071 begins catalysing α1,3-fucosylated HMO once other preferred substrates have been assimilated. Interestingly, when R0071 was co-cultured on the same media with *B. infantis* R0033, notable increases in bacterial cell numbers were observed. This suggests that, despite its transient nature, 3-FL released by R0071, becomes available as a metabolic cross-feeding substrate for other gut commensals, particularly *B. infantis* strains which demonstrate adaptation for uptake and assimilation of the major fucosylated HMO^36^.

Quantification of HMO degradants also revealed that *B. bifidum* R0071 produced sialic acid in stoichiometric accordance with consumption of sialylated HMO. No increase in the levels of lactose was observed after 10 h growth, which demonstrates that *B. bifidum* R0071 hydrolyses HMO in order to access the disaccharide moieties (such as lactose and lacto-*N*-biose) which it then internalises and utilizes for growth. Findings from previous studies suggest that extracellular breakdown of HMO and subsequent liberation of monosaccharides by *B. bifidum* strains can facilitate cross-feeding within microbial communities^37–39^. In particular, studies have highlighted the existence of syntrophic relationships between *B. bifidum* and *B. breve* strains^38,40^. We hypothesise that release of HMO degradants by *B. bifidum* R0071 facilitated growth of *B. breve* M-16V during co-culture growth, with syntrophy between the strains prevailing in the presence of other infant-derived bifidobacteria. When M-16V was co-cultured with *B. bifidum* R0071 on 3′-SL (without the *B. infantis* strains present) its growth was considerably enhanced. These findings highlight that *B. bifidum,* which is generally known to be the minority species in the early gut microbiota, can have a major influence on the prevalence of other species within the *Bifidobacterium* genus. The significance of *B. bifidum* altruistic behaviour in shared environments has been highlighted previously, with studies showing that *B. bifidum* supplementation results in enhanced HMO consumption and increased dominance of bifidobacteria^35,41^.

Here we demonstrate that all four *Bifidobacterium*, individually, have the metabolic machinery required to capture and metabolize HMO into complex health-promoting metabolites, with enhanced production during co-fermentation. We found detectable levels of lactate, acetate, and formate in all cultures. This has potential implications for infant nutritional and health outcomes since production of these key metabolites is critical to the delivery of fundamental ecosystem services^42–47^. In general, higher bacterial biomass resulted in increased secretion of acetate and lactate and correlated with superior rates of HMO consumption. It has been proposed that a theoretical molar ratio of 1.5 acetate to 1 lactate is produced during efficient metabolism of HMO, although other studies have shown that this theoretical ratio is not always obtained^48^. The variation in the theoretical ratio is explained by the production of other sugar metabolites, such as formate and ethanol, which limit the production of lactate. This theoretical ratio was only achieved during *B. infantis* M-63 and co-culture metabolism of S-HMO. In contrast, inefficient hydrolysis of 2’-FL by *B. infantis* R0033 and *B. breve* M-16V shifted metabolism towards greater acetate production resulting in higher average ratios. Studies have demonstrated that increased levels of acetate and formate allow *Bifidobacterium* with atypical HMO consumption phenotypes to balance their reduction–oxidation levels and produce enough energy to continue growing^49^. Formate is produced when pyruvate is shunted away from lactate production, and towards the production of acetyl-CoA^49^. When more than 50% acetyl-CoA is converted to acetate by *B. longum*, the theoretical formate: acetate ratio is 0.4^50^. This ratio was achieved throughout fermentation with the co-culture, irrespective of carbohydrate source. Here, *B. infantis* R0033 and *B. breve* M-16V surpassed that threshold, suggesting that there is a shift to produce more acetyl-CoA than lactate from pyruvate. It is hypothesised that this metabolic shift to favour formate secretion is to prioritize ATP production through a branch in the bifid shunt. We speculate that by shifting metabolic pathways towards formate production, M-16V and R0033 can generate NADH from NAD^+^ without having to use NADH to reduce pyruvate to lactate, resulting in a net positive NADH.

Unlike glucose, galactose, sialic acid, and *N*-acetylglucosamine derivatives, fucose is not predicted to enter the bifid shunt central metabolic pathway. Instead, it is hypothesized that fucose derivatives enter in a separate fermentative pathway, which results in the production of an equimolar ratio of pyruvate and intermediary metabolite 1,2-PD per mole of fucose consumed^51^. Here it was found that co-fermentation enhanced production of 1,2-PD. During growth on 2′-FL, the co-culture and *B. infantis* M-63 exhibited similar levels of growth but 1,2-PD was only detected in the co-culture system. Therefore, factors other than biomass accumulation may play a role in production of this compound. It is possible that fucose transport is repressed in the presence of high concentrations of efficient substrates. James et al. (2019) putatively linked a LacI-family regulator in *Bifidobacterium kashowanohense* (*fumR*) to fucose metabolism^52^. Subsequently, Dedon et al. (2020) determined that *B. infantis* ATCC 15,697 possesses a homolog of this *fumR* and hypothesised that this putative repressor may restrict fucose metabolism in the presence of other efficiently utilized sugars. Therefore, it is possible that increased competition and demand for lactose, glucose, and galactose during co-culture drives metabolism of fucose and subsequent production of 1,2-PD. Enhanced production of 1,2-PD may also occur as a consequence of the trophic chain from the release of fucose during extracellular metabolism of HMO by *B. bifidum* R0071^53^*.* Cross-feeding based on the metabolite 1,2-PD has been proposed to have an important role in the establishment of trophic interactions among gut symbionts^54,55^. Accordingly, it has been reported that the infant-associated *Eubacterium hallii* metabolizes 1,2-PD to produce short chain fatty acids butyrate, formate, and propionate^56,57^. The shift to prioritize 1,2-PD production may therefore facilitate trophic relationships within the infant gut microbiome.

## Conclusion

Deciphering relationships between early colonizing bacterial species and specific HMO types allows for the development of health-promoting products that more closely resemble natural infant microbiota interactions. The findings presented here contribute to our knowledge of HMO-microbe interactions and demonstrate the potential for synbiotic combinations of pre- and pro- biotics. The work demonstrates that small differences in the molecular structure of HMO can have considerable impacts on their biological efficacy and on the catalytic ability of bifidobacterial strains. The variances in HMO metabolism observed in this study highlight the importance of probiotic strain specificity for efficient metabolism of HMO. Genomic and proteomic analyses are required to further understand the functional implications of genotypic variations in HMO-utilization clusters. Development of “HMObiotic” or HMO-containing probiotic products should account for these variations and combine HMO with their preferred pairing of strains. Overall, we conclude that genotype, but also phenotype, of strains should be considered when selecting a strain for probiotic use, as variations in HMO utilization may determine fitness and performance in the gut. We demonstrate the potential benefits for the use of multi-strain bifidobacterial probiotics since co-operation between strains may expand HMO acquisition capabilities and help shift bacterial communities closer towards those observed in breast-fed infants.

## Materials and methods

### Bacterial strains and propagation

*Bifidobacterium* strains used in this study were obtained from Lallemand Health Solutions, France (*B. bifidum* [R0071] and *B. infantis* [R0033]) and Morinaga Milk, Japan (*B. breve* [M-16V] and *B. infantis* [M-63]). All strains were stored at − 80 °C in de Man-Rogosa-Sharpe (MRS) broth (Difco, BD, Ireland), supplemented with 50% (v/v) glycerol as a cryoprotectant. For routine experiments, strains were grown at 37 °C under anaerobic conditions using MRS medium supplemented with 0.05% (wt/v) L-cysteine hydrochloride (Sigma, St. Louis, MO) and 0.01% (wt/v) mupirocin (Merck, Darmstadt, Germany). Bacterial strains were routinely validated using bifidobacterial-specific PCR targeting the 16S region using primers g-Bifid-F (5′-CTCCTGGAAACGGGTGG-3’) and g-Bifid-R (5′-CTCCTGGAAACGGGTGG-3’)^58^.

### Carbohydrate substrates

#### Generation of human milk oligosaccharide enriched fraction

Oligosaccharide fractions were isolated from human milk as previously described^59^. In short, pooled human milk samples (of unknown secretor status) were kindly donated by Irvinestown Human Milk Bank (Co. Fermanagh, Ireland) and stored at − 80 °C on arrival. Lipids and proteins were removed before applying samples to a BioGel P2 size exclusion column (92 × 5 cm; Bio-Rad Laboratories, Inc., Hercules, CA, USA) to separate lactose and HMO components. High pH anion exchange chromatography with pulsed amperometric detection (HPAEC-PAD) was used, as described below, to quantify lactose in the eluted fractions while a Pierce colorimetric peptide assay (Thermo Fisher Scientific, Reinach, Switzerland) was used to measure levels of peptides. Peptide-free and low-trace lactose (< 80 mg/L) fractions were pooled and freeze-dried and the resultant HMO-enriched fraction was stored at 4 °C prior to use in experiments.

#### Preparation of HMO blends

Three HMO powders were assessed in this study; a HMO-enriched fraction with and without 2′-FL (Friesland Campina, Amersfoort, Netherlands) was used to mimic non-secretor (NS-HMO) and secretor (S-HMO) human milk oligosaccharide fractions, respectively. 2′-FL alone was used as a control (Table 2). Stock suspensions (20% wt/v) of 2′-FL, NS-HMO, and S-HMO were prepared in MQ-water and sterile filtered (0.2 µm, Chromafil, Macherey–Nagel, Düren, Germany) Oligosaccharide substrates were used in fermentation studies at concentrations outlined in Table 2. The maximum levels of 2′-FL authorised according to the Official Journal of the European Union^60^ is 1.2 g/L while preliminary studies indicated that 3.8 g/L was the lowest concentration of HMO that resulted in excess oligosaccharide in culture system after 24 h bacterial fermentation (data not shown).

**Table 2:** Concentrations of HMO used in fermentation experiments.

	2′-FL Control	NS-HMO	S-HMO
2′-fucosyllactose (g/L)	1.2	–	1.2
Isolated HMO (g/L)	–	3.8	3.8

### Pure culture and co-culture fermentations

#### Growth medium

Modifications were made to Man-Rogosa-Sharpe (MRS)^61^ medium to generate a basal growth medium which contained no carbohydrate sources as previously described^62^. Media was sterilized by autoclaving at 121 °C for 15 min. For use in fermentation studies, cfMRS medium was supplemented with sterile oligosaccharide solutions at concentrations outlined in Table 2.

#### Preparation of inoculum and initiation of fermentation process

For experiments, bacteria derived from −80 °C stock cultures were streaked on MRS agar supplemented with 0.05% (wt/v) L-cysteine HCl and 0.01% (wt/v) mupirocin and incubated for 48 h at 37 °C under anaerobic conditions. Single colonies of each strain were subsequently inoculated in supplemented MRS broth, and subcultured twice before use in experiments. To obtain working cultures, supernatants of overnight media was removed, the bacterial cell pellet washed with Phosphate Buffer Saline (PBS) (Merck), and resuspended in cfMRS such that the OD_600_ of each bacterial culture was ~ 2. The following bacterial combinations were assessed: single cultures of *B. bifidum* (R0071), *B. infantis* (R0033), *B. breve* (M-16V), *B. infantis* (M-63) and a co-culture of all four *Bifidobacterium* (4Bif). Blanks of uninoculated media were also included. Ten milli-litres of oligosaccharide supplemented media (2′-FL, NS-HMO, S-HMO, or cfMRS) was inoculated with 1% (v/v) of overnight cultures to initiate fermentations. All cultivations were carried out under anaerobic conditions at 37 °C.

More in-depth analysis of potential cross-feeding relationships was provided by performing mono- and co-culture fermentations of (a) *B. bifidum* R0071 and *B. infantis* R0033 on 5 mg/mL DFL and (b) *B. bifidum* R0071 and *B. breve* M-16V on 5 mg/mL 3′-SL. Cultures were prepared as described above. At 0, 10, and 24 h bacterial cultures were serially diluted in Maximum Recovery Diluent (Oxoid) and enumerated by plating on MRS agar. Bacteria was quantified according to the formula:

$${\text{cfu}}/{\text{mL}} = \frac{{{\text{no}}{.} \;{\text{of}}\; {\text{colonies}}\; \times \;{\text{dilution}}\; {\text{factor}}}}{{{\text{volume}}\;{\text{ of}}\;{\text{ culture}}\;{\text{ plated}}}}$$

Definitive differences in colony characteristics between R0071 & R0033, and R0071 & M-16V allowed for strain-level quantification during co-cultivation of strains.

### Growth analysis

#### Optical density readings

Variability in bacterial biomass was assessed throughout fermentation by measuring optical density at 600 nm (OD_600 nm_). Growth analysis was performed on cultures at 0 h, 6 h (early exponential), 10 h (late exponential), 20 h (early stationary), and 24 h (late stationary). At each time point, 200 µl of culture was transferred to the wells of 96 well microtiter plates in an anaerobic chamber and the OD values determined using a Synergy 2 plate reader (BioTek Instruments, Inc., Vermont, US). Turbidity of cultures was assumed to correlate with numbers of bacterial cells within solution.

#### Quantitative PCR

Possible enhancement or reduction of bifidobacterial growth as a consequence of co-cultivation with other strains was evaluated through absolute quantification of bacterial numbers by quantitative PCR (qPCR). One ml samples were harvested from cultures at 0 h, 10 h, and 24 h. Bacterial cells were pelleted at 12,000×*g* for 2 min prior to the addition of the lysis buffer. Total bacterial DNA was isolated using GenElute Bacterial Genomic DNA Kit (Merck) according to manufacturer’s instructions. The resultant DNA was quantified by a Nanodrop 2000 Spectrophotometer. Total bifidobacterial numbers were quantified on a 480 Lightcycler platform (Roche Applied Science, Penzberg, Germany) using the following programme: 95 °C for 5 min followed by 40 cycles of 95 °C for 10 s, 58 °C for 20 s and 72 °C for 30 s. The fluorescent product was detected at the last step of each cycle. Following amplification, melting temperature analysis of PCR products was performed to determine the specificity of the PCR. The melting curves were obtained by slow heating at 0.2 °C/s increments from 60 to 99 °C, with continuous fluorescence collection. Reactions took place in a 25 µl volume made up of 5 µl PCR grade water, 1.25 µl forward primer (0.10 µM), 1.25 µl reverse primer (0.10 µM), 5 µl DNA template and 12.5 µl SYBR green (Roche Diagnostics, West Sussex, United Kingdom). Experiments were based on previously designed strain-specific primers targeting the 16 s gene region. For quantification of all *Bifidobacterium* strains present in samples, the primer pairs Bifid-F (CTCCTGGAAACGGGTGG) and g-Bifid-R (GGTGTTCTTCCCGATATCTACA) were used. To determine *B. bifidum* cell numbers, primer pairs BiBIF-1 (5′-CCACATGATCGCATGTGATTG-3′) and BiBIF-2 (5’-CCGGATGCTCCATCACAC-3’) were used. *B. breve* cell numbers were quantified using primer pairs BiBRE-1 (5′-CCACATGATCGCATGTGATTG-3′) and BiBRE-2 (5′-ACAAAGTGCCTTGCTCCCT-3′), while *B. infantis* cell numbers were determined using primer pairs BiINF-1 (5′-TTCCAGTTGATCGCATGGTC-3′) and BiINF-2 (5′- GGAAACCCCATCTCTGGGA -3′).

The copy-number of a given strain in co-culture was evaluated by comparing the cycle threshold (Ct) values with those from a standard curve of known copy number. Standard curves (10^9^–10^2^ copy number/µL) were established by performing 1 in 10 serial dilutions of 16S rDNA of the specified bifidobacterial strain (in the case of *B. infantis* strains, M-63 was used as the reference rDNA). The cell numbers of strains within co-culture (denoted by bifidobacteria/mL) were then deduced by comparing these copy number values with copy numbers of standards with known cell numbers (as determined by viable count assessment). Cell numbers were calculated according to the formula^63^:

$$\frac{{\left( {{\text{C}}/{\text{ul}}} \right)\left( {{\text{TV}}} \right)}}{{{\text{TCN}}}} \times {\text{Tcfu}}/{\text{ml}} = {\text{ bifidobacteria}}/{\text{ml }}\left( {\text{S}} \right)$$

where C/μl = Copy number/μl, TV = template volume, TCN = total copy number of the standard used, T cfu/ml = total cell number of standard used, and bifidobacteria/mL(S) = cell number of test sample. Samples were run in triplicate, while standards and negative controls (where template DNA was replaced with PCR grade water) were run in duplicate.

Growth rate (µ) of each subspecies within cultures was calculated using the following equation:

$$\mu = \frac{{\ln X_{2} - \ln X_{1} }}{{t_{2} - t_{1} }}$$

where *X*_2_ and *X*_1_ are the cell densities at t_2_ and t_1_. Mean doubling time (T_d_) was calculated as:

$$T_{d} = {\raise0.7ex\hbox{${\ln 2}$} \!\mathord{\left/ {\vphantom {{\ln 2} \mu }}\right.\kern-\nulldelimiterspace} \!\lower0.7ex\hbox{$\mu $}}$$

### Chemical analysis of culture supernatants

For chromatographic analysis of cultures, supernatants were extracted at late exponential phase (10 h) and filtered using 0.2 µm nylon syringe filters (Chromafil, Macherey–Nagel, Düren, Germany) to remove bacterial cell debris.

#### Quantification of HMO utilization

The concentrations of oligosaccharide in culture after 10 h microbial fermentation were determined by HPAEC-PAD using a Dionex ICS-3000 Series system (Dionex Corporation, Sunnyvale, CA, USA). Samples were diluted 100-fold with MQ-Water, and 20 µl injected onto a CarboPac PA100 column (250 × 4 mm) (Thermo Fisher Scientific) equipped with a guard column. 100 mM NaOH (A) and 100 mM NaOH plus 500 mM Na-acetate (B) were used as eluents at a flow rate of 1.0 mL/min. The column temperature and detector were maintained at 32 °C. HMO were separated for 22 min using an eluent gradient as follows: 95% A and 5% B for 3 min, 88% A and 12% B for 10 min, 84% A and 16% B for 4 min and hold time of 3 min, followed by 82% A and 18% B for 2 min.

At 22.1 min the gradient immediately changes to 50% A and 50% B for 8 min to regenerate the column and then back to 95% A and 5% B for a further 15 min to re-equilibrate the column. The eluent was monitored by pulsed amperometric detection (PAD). The eluted sugars were identified from their elution times relative to those of external standards: Lactose, 3-FL, 2′-FL, LNnT, LNnH, LNT, LNH, sialic acid, sialyllacto-*N*-tetraose a (LSTa), LSTc, 6′-SL, 3′-SL, disialyllacto-*N*-tetraose (DSLNT). Utilization of HMO was calculated as % consumption versus the uninoculated control.

#### Quantification of organic acid metabolites

To examine organic acid production, supernatants were extracted after 10 h fermentation and filtered as above. Levels of organic acid metabolites were then quantified by HPLC using a Waters Alliance Separations module e2695 coupled to a Waters 2414 refractive index (RI) detector (Waters, Milford MA, USA). Samples or standards at a volume of 20 µl were injected on to an Aminex HPX 87C fixed ion resin column (300 × 7.8 mm) operated at 60 °C. The samples were eluted with H_2_SO_4_ (0.005 N) at a flow rate of 0.6 mL/ min. Sample detection was performed by comparing retention times of standards. Analytical grade acetic acid, lactic acid, and formic acid supplied by Merck were used as standards.

#### Quantification of 1,2-propanediol

To assess whether co-culture fermentation of HMO increased 1,2-PD production when compared to single culture fermentation, samples were sent to MS-Omics ApS, Frederiksberg, Denmark for analysis using GC-MS^64^. A mixed pooled sample (QC sample) was created by taking an aliquot from each sample. This sample was analyzed with regular intervals throughout the sequence. For 1,2-PD analysis, samples were acidified using hydrochloric acid, and deuterium-labeled internal standards were added. Analysis was performed using a high-polarity column (ZebronTM ZB-FFAP, GC Cap. Column 30 m × 0.25 mm × 0.25 μm) installed in a GC (7890B, Agilent) coupled with a quadrupole detector (59977B, Agilent). Raw data was converted to netCDF format using Chemstation (Agilent), before the data was imported and processed in Matlab R2014b (Mathworks, Inc.) using the PARADISe software.

## Statistical analysis

Statistical analysis was calculated using technical triplicate data from biological triplicate experiments. Graphs were created using in PRISM v8 (GraphPad, La Jolla, CA). Univariate analysis of variance (ANOVA) and post-hoc Tukey tests were performed to determine the significant differences between the groups for growth, oligosaccharide consumption and metabolite production. Statistical significance was accepted as *p* ≤ 0.05.

## Supplementary Information

Supplementary Information.
