# Supplementary material for: Human milk oligosaccharide-sharing by a consortium of infant derived Bifidobacterium species
Source: Sci Rep. 2022 Mar 9;12:4143. doi: 10.1038/s41598-022-07904-y (PMC8907170; doi:10.1038/s41598-022-07904-y)
Supplement: Supplementary file 1 — Supplementary Information. [file 41598_2022_7904_MOESM1_ESM.docx]

**SUPPLEMENTARY FIGURE 1:**


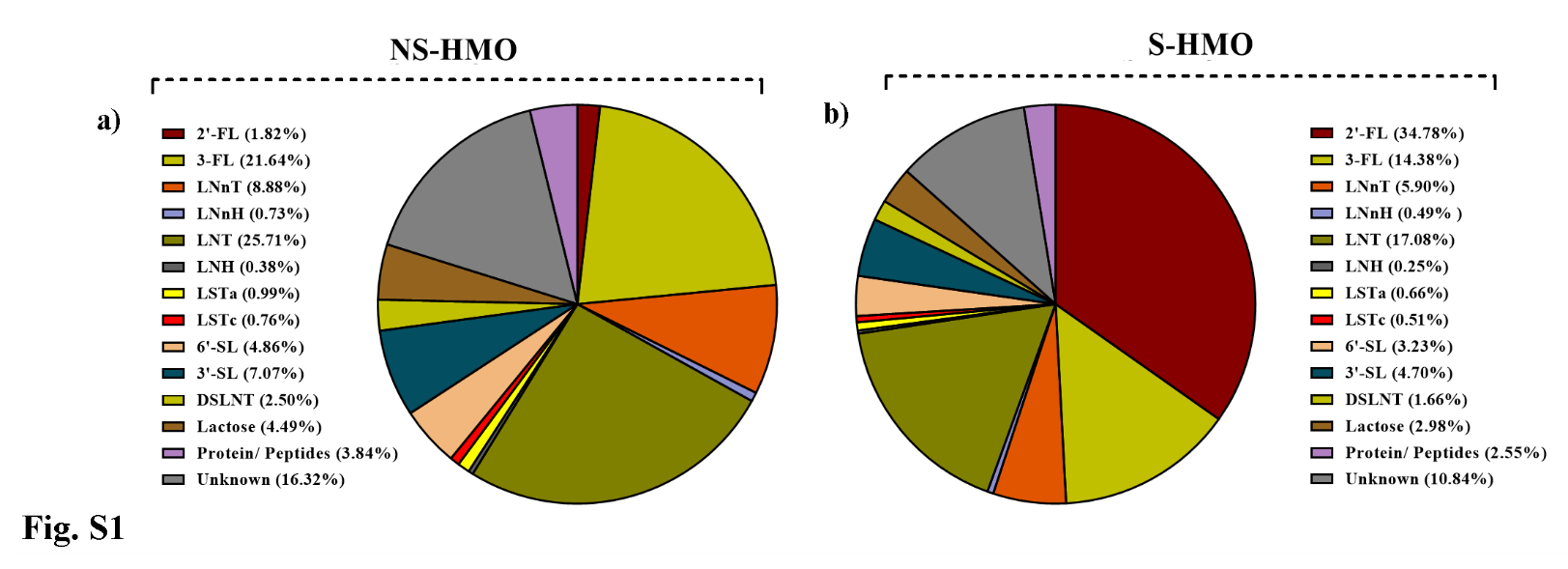


The pie charts show the relative abundance of the major oligosaccharides found in a) NS-HMO and
b) S-HMO which were detected via HPAEC-PAD analysis by comparing to the following external HMO standards: lactose, 3-fucosyllactose (3-FL), 2’-fucosyllactose (2'-FL), lacto-*N*-neotetraose (LNnT), lacto-*N*-neohexaose (LNnH), lacto-*N*-tetraose (LNT), lacto-*N*-hexaose (LNH), sialyllacto-*N*-tetraose a (LSTa), sialyllacto-*N*-tetraose a (LSTc), 6’-siallylactose (6'-SL), 3’-siallylactose (3'-SL), disialyllacto-*N*-tetraose (DSLNT). Data represents average concentrations of technical duplicate data from biological triplicate experiments.

**SUPPLEMENTARY FIGURE 2:**


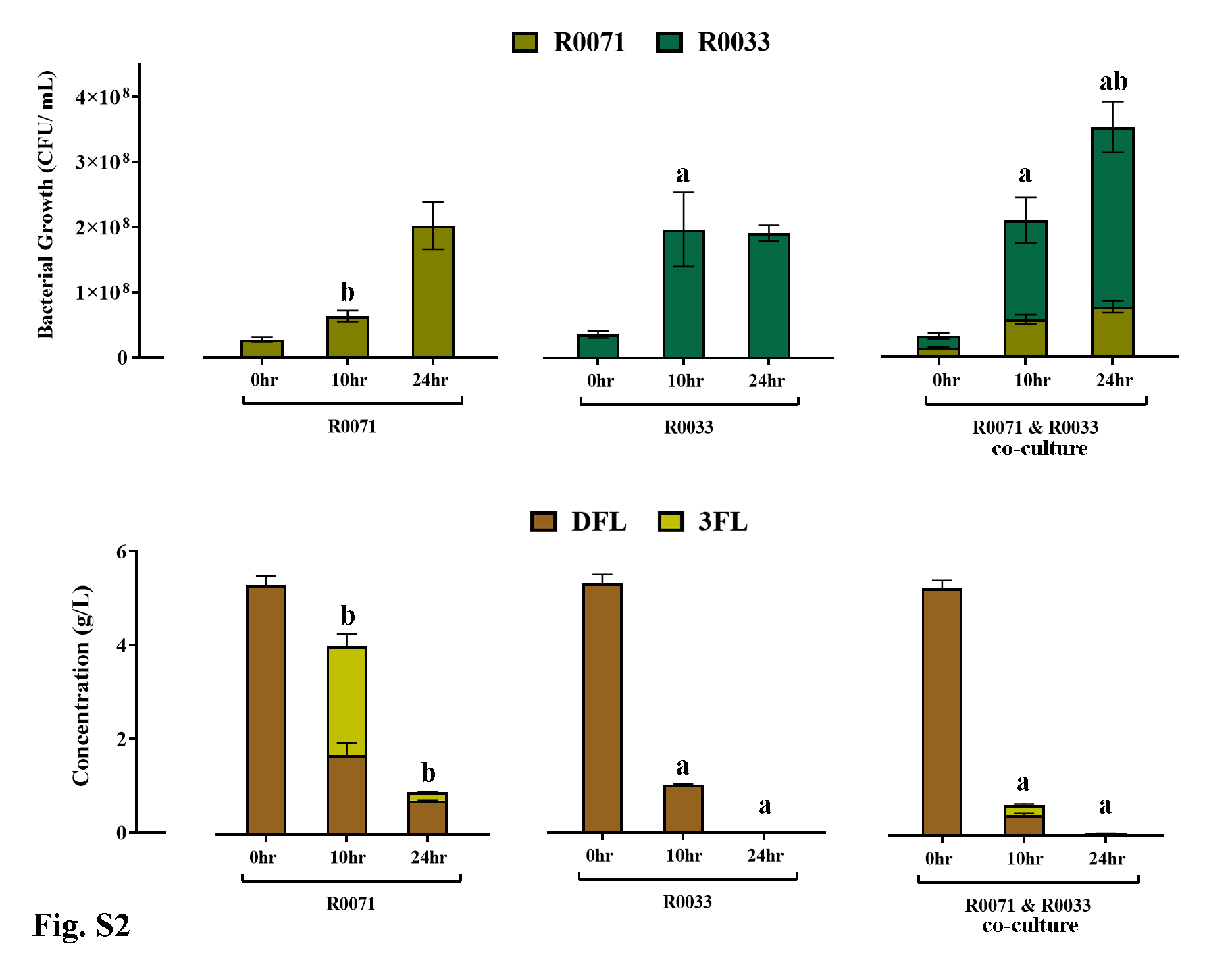


Growth profiles in CFU/mL (top) and glycoprofiles in g/L HMO (bottom) of *Bifidobacterium bifidum* R0071 and *Bifidobacterium infantis* R0033 as mono-cultures and as a two strain co-culture with 5g/L difucosyllactose (DFL) as the sole carbon source. Bacterial cell numbers were assessed using colony counts at 0 hr, 10 hr, and 24 hr. Definitive differences in colony characteristics between R0071 & R0033 allowed for strain-level quantification during co-cultivation of strains. The concentrations of oligosaccharides in the culture supernatants was determined by HPAEC-PAD. The sugars were identified from their elution times relative to those of external standards. Analysis was calculated using technical triplicate data from biological triplicate experiments and data are means +/- SD. Univariate analysis of variance (ANOVA) and post-hoc Tukey tests were performed to determine the significant differences between the groups (a = *p* <0.05 versus R0071 mono-culture, b = *p* <0.05 versus R0033 mono-culture)

**SUPPLEMENTARY FIGURE 3:**


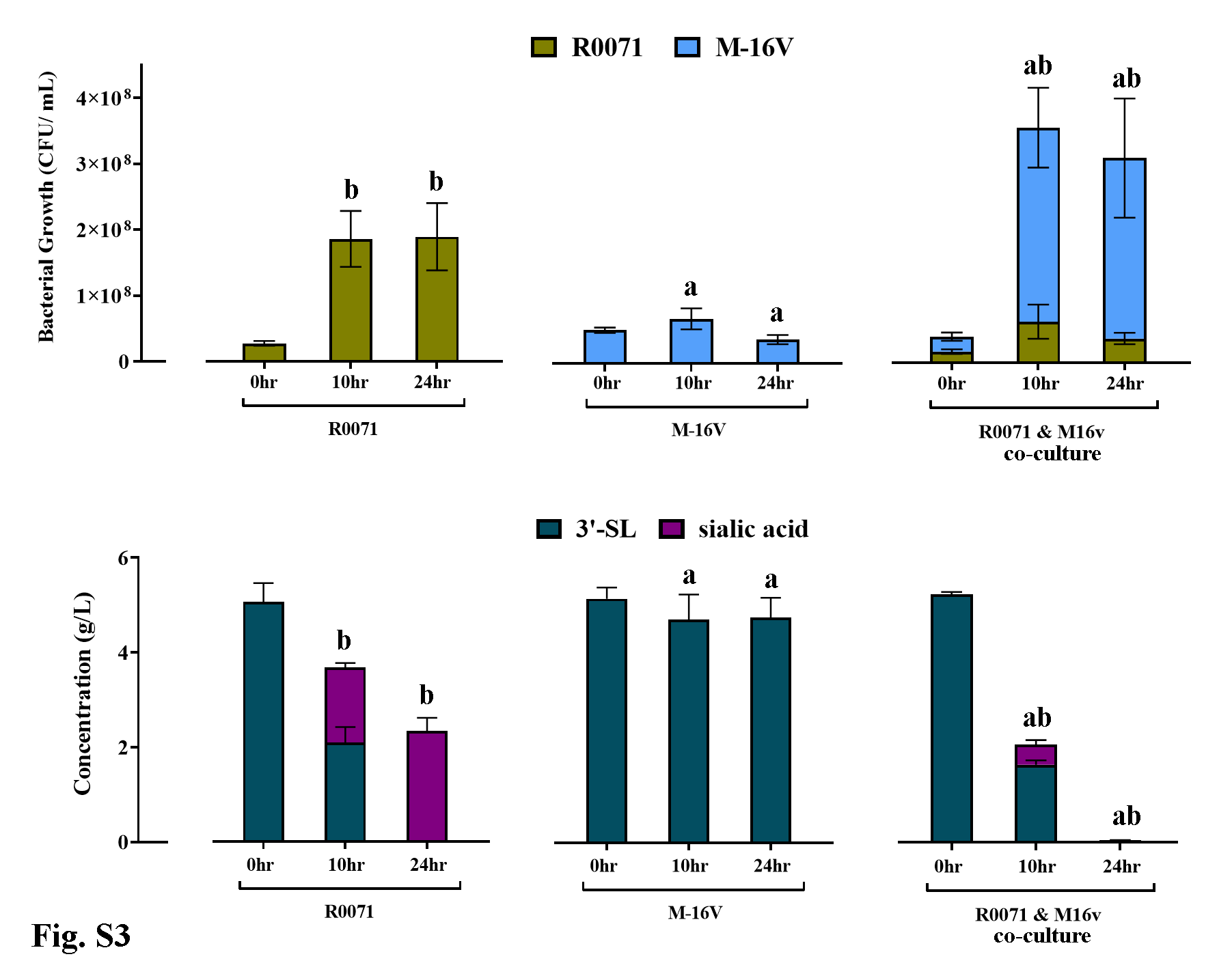


Growth profiles in CFU/mL (top) and glycoprofiles in g/L HMO (bottom) of *Bifidobacterium bifidum* R0071 and *Bifidobacterium breve* M-16V as mono-cultures and as a two strain co-culture with 5g/L 3’-siallylactose (3’-SL) as the sole carbon source. Bacterial cell numbers were assessed using colony counts at 0 hr, 10 hr, and 24 hr. Definitive differences in colony characteristics between R0071 & M-16V allowed for strain-level quantification during co-cultivation of strains. The concentrations of oligosaccharides in the culture supernatants was determined by HPAEC-PAD. The sugars were identified from their elution times relative to those of external standards. Analysis was calculated using technical triplicate data from biological triplicate experiments and data are means +/- SD. Univariate analysis of variance (ANOVA) and post-hoc Tukey tests were performed to determine the significant differences between the groups (a = *p* <0.05 versus R0071 mono-culture, b = *p* <0.05 versus M-16V mono-culture)

**SUPPLEMENTARY FIGURE 4:**


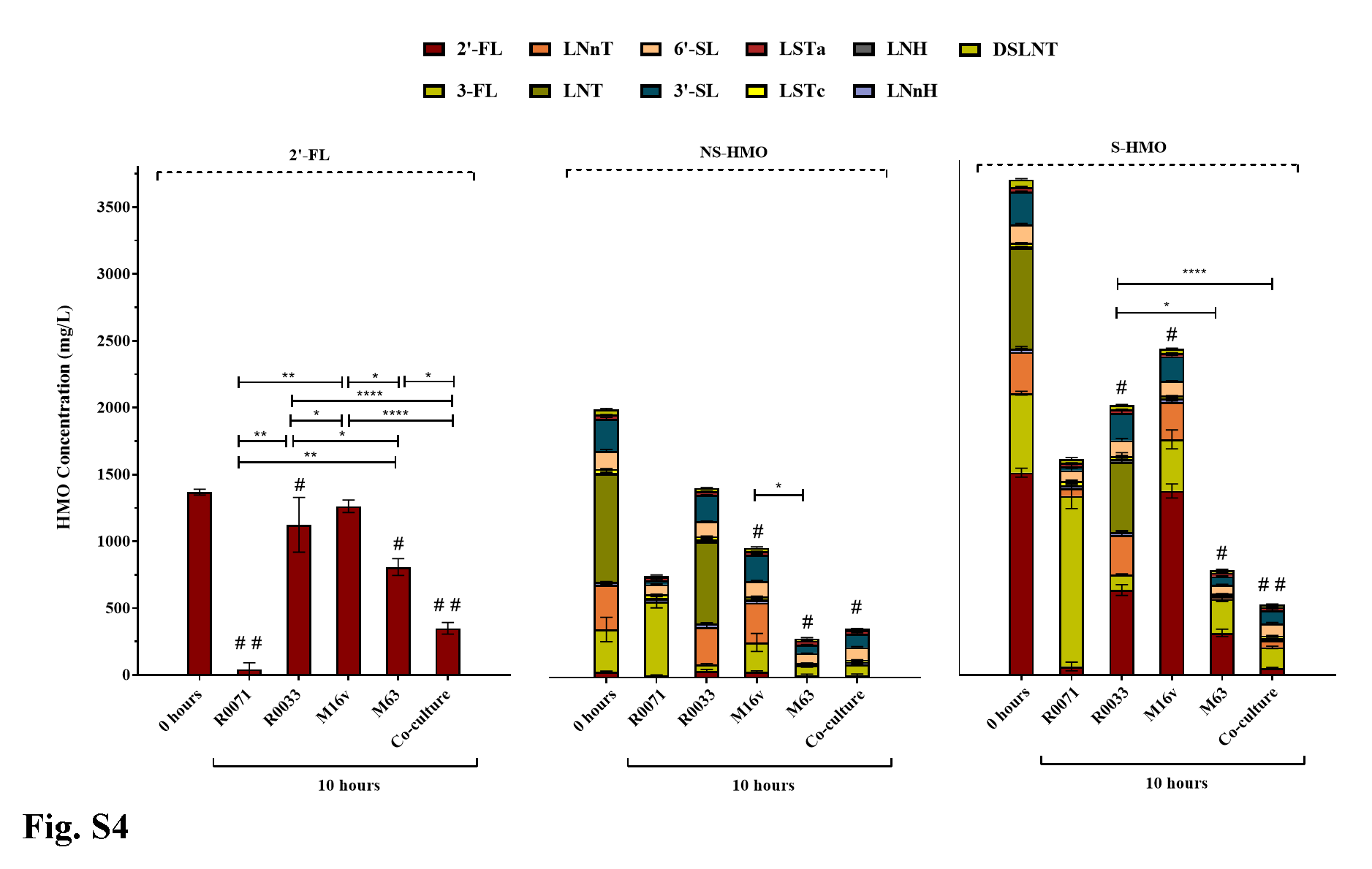


Concentration of HMO (2’-FL, NS-HMO and S-HMO) before and after 10 hours of bifidobacterial fermentation detected via HPAEC-PAD analysis by comparing to the following external HMO standards: 3-fucosyllactose (3-FL), 2’-fucosyllactose (2'-FL), lacto-*N*-neotetraose (LNnT), lacto-*N*-neohexaose (LNnH), lacto-*N*-tetraose (LNT), lacto-*N*-hexaose (LNH), sialyllacto-*N*-tetraose a (LSTa), sialyllacto-*N*-tetraose a (LSTc), 6’-siallylactose (6'-SL), 3’-siallylactose (3'-SL), disialyllacto-*N*-tetraose (DSLNT). R0071= *Bifidobacterium bifidum* R0071, R0033= *Bifidobacterium infantis* R0033, M-16V= *Bifidobacterium breve* M-16V, M-63= *Bifidobacterium infantis* M-63. Analysis was calculated using technical duplicate data from biological triplicate experiments and data are means +/-SD. Univariate analysis of variance (ANOVA) and post-hoc Tukey tests were performed to determine the significant differences between the groups (# = *p* <0.05, ## = *p* <0.001 versus Time 0) (* = *p* <0.05, ** = *p* <0.01, *** = *p* <0.001, **** = *p* <0.0001 between strain combinations).

**SUPPLEMENTARY FIGURE 5**:


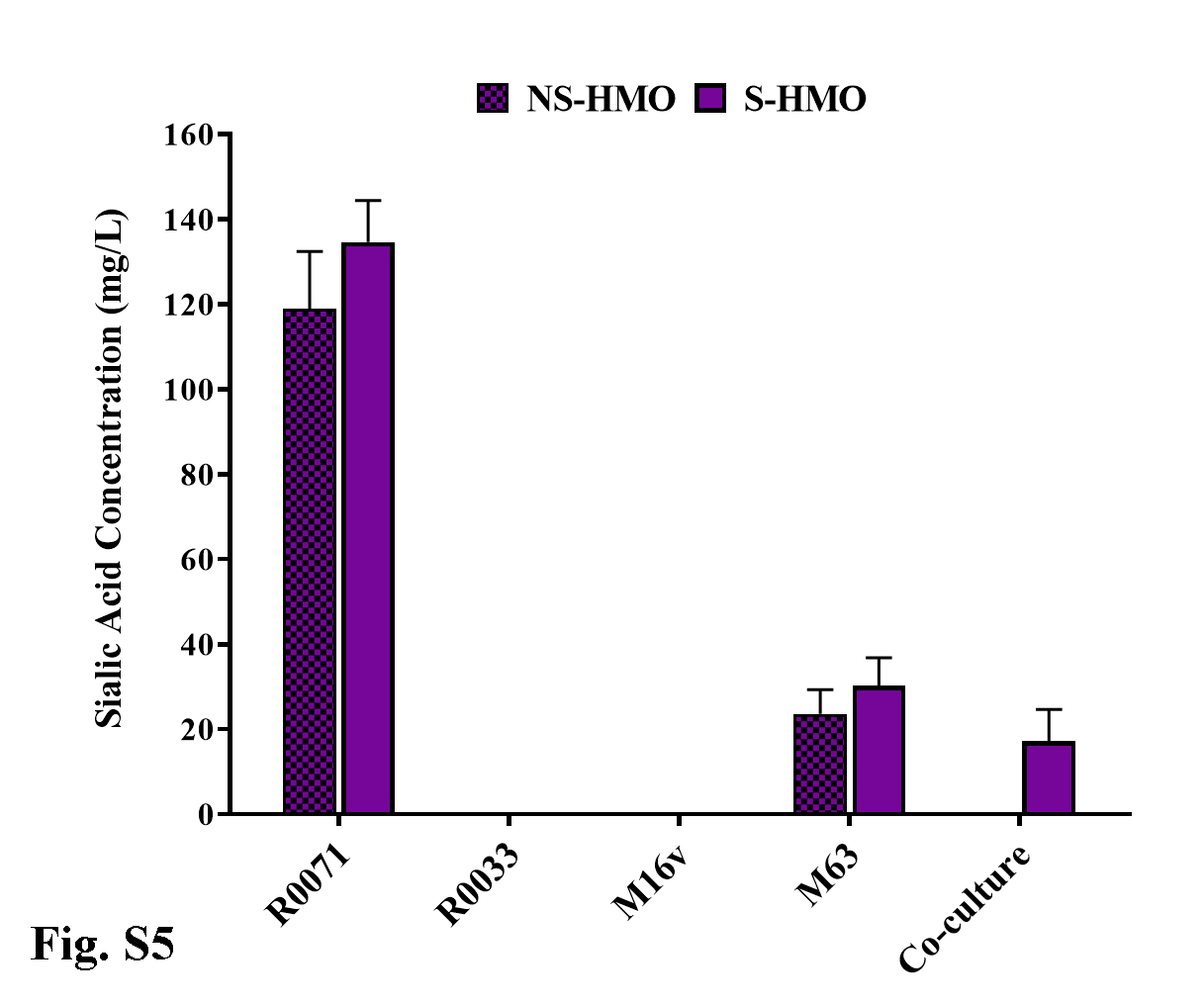


Quantification of free sialic acid following 10 hours of bifidobacterial fermentation on NS-HMO and S-HMO detected via HPAEC-PAD analysis by comparing to an external sialic acid standard. R0071= *Bifidobacterium bifidum* R0071, R0033= *Bifidobacterium infantis* R0033, M-16V= *Bifidobacterium breve* M-16V, M-63= *Bifidobacterium infantis* M-63. Analysis was calculated using technical duplicate data from biological triplicate experiments and data are means +/-SD.

**SUPPLEMENTARY FIGURE 6**:

**
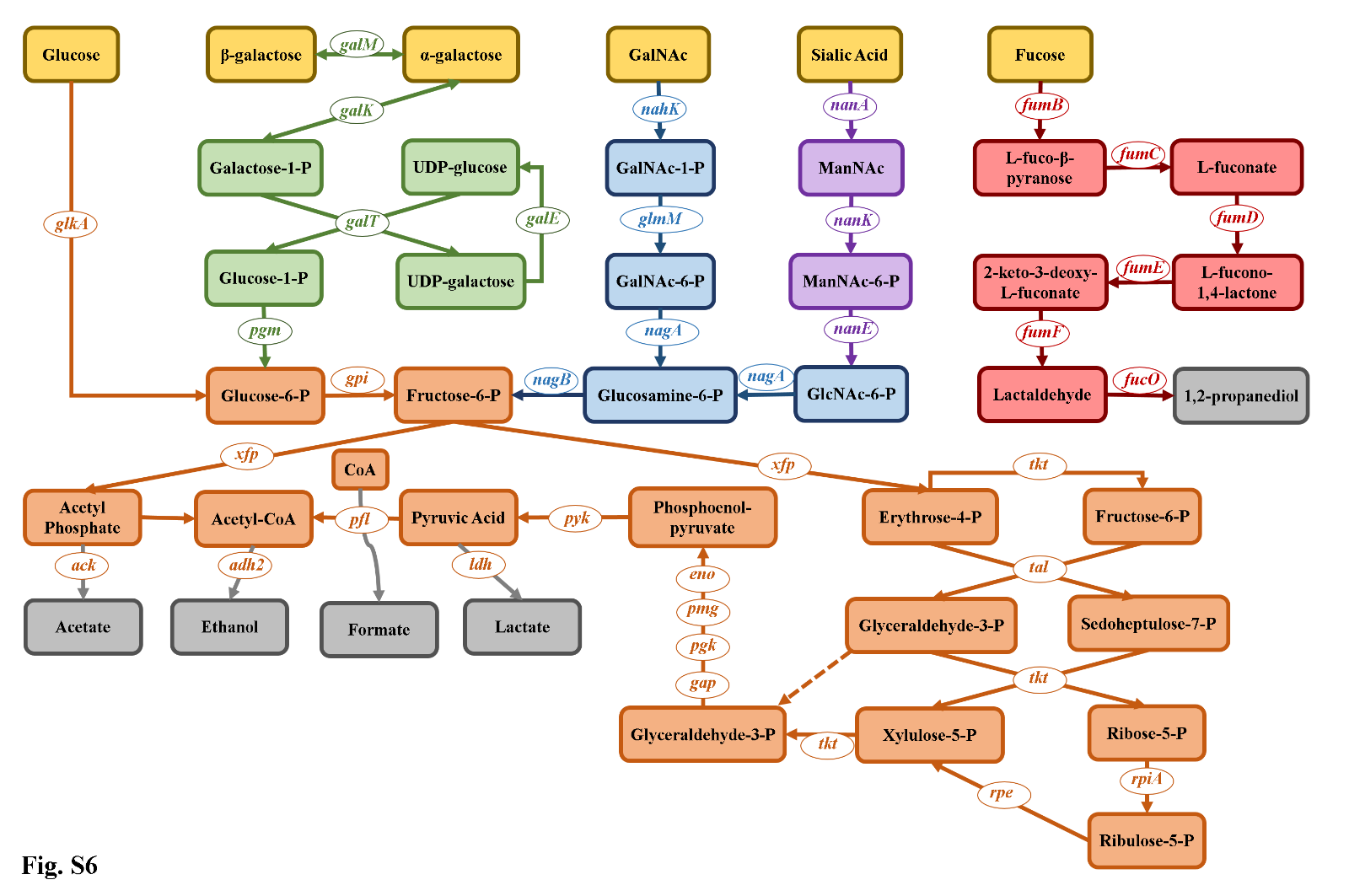
**

**A schematic representation of carbohydrate degradation through the “bifid shunt” and related pathways in bifidobacteria.** Gene names: Leloir genes (green): *galM* galactose mutarotase; *galK* galactokinase; *galT* galactose-1-phosphate uridylyltransferase; *pgm* phosphoglucomutase. Central fermentative pathway genes (orange): *glkA* glucokinase; *gpi* glucose-6-phosphate isomerase; *xfp* fructose-6-phosphate phosphoketolase/ xylulose-5-phosphate phosphoketolase; *tkt* transketolase; *tal* transaldolase; *rpiA* ribose 5-phosphate isomerase; *rpe* ribulose 5-phosphate epimerase; *gap* glyceraldehyde-3-phosphate dehydrogenase; *pgk* phosphoglycerate kinase; *pmg* phosphoglycerate mutase; *eno* enolase; *pyk* pyruvate kinase; *ack* acetate kinase; *adh2* aldehyde-alcohol dehydrogenase; *pfl* formate acetyltransferase; *ldh* lactate dehydrogenase. *N-*acetylglucosamine genes (blue): *nahK* *N-*acetylhexosamine 1-kinase; *GlmM* phosphoglucosamine mutase; *nagA* *N-*acetylglucosamine-6-phosphate deacetylase; *nagB* glucosamine-6-phosphate isomerase. Sialic Acid genes (purple): *nanA* *N-*acetylneuraminate lyase; *nanK* *N-*acetylmannosamine kinase; *nanE* *N-*acetylmannosamine-6-phosphate 2-epimerase. Fucose genes (red) *(as proposed by Dedon et al. 2020)*: *fumB* L-fucose mutarotase; *fumC* L-fucose dehydrogenase; *fumD* L-fuconolactone hydrolase; *fumE* L-fuconate dehydratase; *fumF* aldolase; *fumO* lactaldehyde reductase. Monosaccharide HMO derivatives depicted in yellow. End metabolites depicted in grey.

| Supplementary Table 1: Organic Acid Concentrations | | | | | | | | | | | | | | | | | |
| --- | --- | --- | --- | --- | --- | --- | --- | --- | --- | --- | --- | --- | --- | --- | --- | --- | --- |
|  |  | **Acetate (mM)** | | |  | **Lactate (mM)** | | |  | **Formate (mM)** | | |  | **1,2-propanediol (mM)** | | |  |
|  |  | 2'-FL | NS-HMO | S-HMO |  | 2'-FL | NS-HMO | S-HMO |  | 2'-FL | NS-HMO | S-HMO |  | 2'-FL | NS-HMO | S-HMO |  |
| *Bbif* R0071 |  | 4.6 ± 1.4 | 19.7 ± 5.4 | 24.4 ± 0.8 |  | 3.5 ± 0.6 | 8.5 ±  0.2 | 12.7 ± 0.9 |  | 2.9 ± 0.2 | 6.7 ±  0.2 | 9.0 ± 1.4 |  | <LOD | <LOD | <LOD |  |
| *BInf* R0033 |  | 3.9 ± 1.8 | 3.1 ±  1.0 | 11.6 ± 3.3 |  | 0.2 ± 0.1 | 1.0 ±  0.2 | 5.6 ± 1.0 |  | 2.7 ± 0.2 | 3.2 ±  0.9 | 6.7 ± 0.8 |  | <LOD | <LOD | 2.4 ±  0.3 |  |
| *Bbrev* M-16V |  | 2.4 ± 0.8 | 7.2 ±  2.0 | 6.9 ± 2.1 |  | 0.2 ± 0.2 | 1.0 ±  0.3 | 2.3 ± 0.7 |  | 4.0 ± 0.7 | 5.3 ±  1.5 | 4.3 ± 0.5 |  | <LOD | <LOD | <LOD |  |
| *Binf* M-63 |  | 7.8 ± 2.2 | 19.9 ± 1.5 | 23.1 ± 4.5 |  | 1.4 ± 0.2 | 15.5 ±  0.5 | 14.9 ± 12.7 |  | 2.8 ± 0.5 | 3.3 ±  1.2 | 5.4 ± 1.2 |  | <LOD | 2.1 ±  0.3 | 2.1 ±  0.6 |  |
| 4Bif co-culture |  | 11.6 ± 5.5 | 23.6 ± 3.1 | 25.6 ± 2.1 |  | 3.2 ± 0.1 | 13.9 ± 1.1 | 17.3 ± 1.7 |  | 5.3 ± 1.2 | 7.2 ±  1.8 | 8.1 ± 0.5 |  | 1.7 ± 0.0 | 2.4 ±  0.4 | 3.0 ±  0.3 |  |
| <LOD= below the limit of detection | | | | | | | | | | | | | | | | | |
